# A New Cutting Tool Design for Cryogenic Machining of Ti–6Al–4V Titanium Alloy

**DOI:** 10.3390/ma12030477

**Published:** 2019-02-04

**Authors:** Alborz Shokrani, Stephen T Newman

**Affiliations:** Department of Mechanical Engineering, University of Bath, BA2 7AY Bath, UK; s.t.newman@bath.ac.uk

**Keywords:** cryogenic machining, cutting tool, cutting geometry, titanium

## Abstract

Titanium alloys are extensively used in aerospace and medical industries. About 15% of modern civil aircrafts are made from titanium alloys. Ti–6Al–4V, the most used titanium alloy, is widely considered a difficult-to-machine material due to short tool life, poor surface integrity, and low productivity during machining. Cryogenic machining using liquid nitrogen (LN_2_) has shown promising advantages in increasing tool life and material removal rate whilst improving surface integrity. However, to date, there is no study on cutting tool geometry and its performance relationship in cryogenic machining. This paper presents the first investigation on various cutting tool geometries for cryogenic end milling of Ti–6Al–4V alloy. The investigations revealed that a 14° rake angle and a 10° primary clearance angle are the most suitable geometries for cryogenic machining. The effect of cutting speed on tool life was also studied. The analysis indicated that 110 m/min cutting speed yields the longest tool life of 91 min whilst allowing for up to 83% increased productivity when machining Ti–6Al–4V. Overall the research shows significant impact in machining performance of Ti–6Al–4V with much higher material removal rate.

## 1. Introduction

Titanium is one of the most desirable materials in engineering applications where weight is a major concern. It has the highest strength-to-weight ratio amongst all structural materials and can withstand high temperatures. Grade 5 titanium, Ti–6Al–4V, with α-β microstructure is one of the most used titanium alloys in industry, forming more than 50% of the global production [1]. Due to its inherent mechanical properties, titanium alloys are extensively used in aerospace industries. It is reported that 14% of the Airbus A350-900 [2] and 15% of Boeing 787 [3] aeroframes are made of titanium alloys. Manufactured from wrought material, investment casting or even additive manufacturing, all parts require machining processes for finishing in order to achieve the required surface finish, engineering tolerances, and mechanical properties such as predictable fatigue life.

The material properties that make titanium alloys an attractive material for many engineering applications are also responsible for making them difficult-to-machine materials [4]. Machining titanium is often associated with short tool life, poor surface integrity, and low productivity [5]. Due to the high material strength and poor thermal conductivity, high temperatures are generated during machining titanium alloys [6]. High temperatures at the cutting zone can result in thermal softening of the cutting tool material and increase chemical reaction between the cutting tool and workpiece materials leading to diffusion and adhesion wear. In recent years, significant research has been conducted to improve the machinability of titanium alloys [5,6,7,8,9,10]. One of the methods which has attracted many researchers is cryogenic machining using liquefied gases such as liquid nitrogen (LN_2_) and liquid carbon dioxide (CO_2_) [11,12,13]. In this method, a liquefied gas at very low temperature is sprayed over the cutting zone to cool the workpiece, the cutting tool and the cutting zone. Super cold liquefied gases can favorably alter the material properties of the workpiece and/or cutting tool and enhance heat removal from the cutting zone [14,15].

Investigations by various researchers have shown that cryogenic cooling can enhance the machinability of difficult-to-machine materials by extending tool life and improving surface finish [16,17,18,19]. Lee et al. [20] reported that using cryogenic cooling with LN_2_ and a preheated Ti–6Al–4V workpiece can result in up to 90% increased tool life. Similarly, Park et al. [21] reported that using LN_2_ coolant resulted in reduced tool wear when face milling Ti–6Al–4V alloy. Pereira et al. [12] compared the impact of different machining environments in turning AISI 304 stainless steel. In their study, the effect of LN_2_ and CO_2_ as well as combined cryogenic cooling and minimum quantity lubrication (MQL) on tool life, cutting forces and surface integrity were investigated. They noted that using CO_2_ as a cryogen performs better than LN_2_ coolant when turning AISI 304. The addition of MQL lubrication further improved machinability irrespective of the cryogen used [12]. In a similar study [22], the impact of various cooling and lubrication scenarios were tested in turning Ti–6Al–4V. Iqbal et al. [22] reported that the lowest tool wear was associated with combined LN_2_ cryogenic cooling and MQL followed by LN_2_ cryogenic machining and combined CO_2_ and MQL. This indicated that the effect of cryogenic cooling and MQL depends on the workpiece and cutting tool material pair and setup as identified by Zhao and Hong [23].

Rotella et al. [24] investigated the effect of various cooling conditions on surface finish of Ti–6Al–4V alloy in turning operations. They noted that cryogenic cooling significantly improved the surface finish of the machined parts in terms of surface roughness and surface hardness. Bordin et al. [25,26] studied the effect of cryogenic cooling in machining additively manufactured Ti–6Al–4V alloy. The investigations indicated that cryogenic cooling can inhibit adhesion wear and reduce the tool-chip contact length and concluded that increasing feed rate and cutting speed results in higher tool wear. The investigations also indicated that improved surface roughness can be achieved using cryogenic cooling. Pereira et al. [27] proposed a nozzle design based on the Coanda effect for combined cryogenic cooling with CO_2_ and MQL. The nozzle was tested in turning Inconel 718 alloy and compared with flood cooling. Using only CO_2_ cryogenic cooling, the tool life was only 68% that of flood cooling. The addition of MQL to the cryogenic cooling increased the tool life to 93% of the tool life achieved with flood cooling.

A number of researchers studied the effect of cryogenic delivery methods on the machining performance by developing new cutting tool designs or modifying existing tools [28]. Hong et al. [29], Dhananchezian and Kumar [30], and Bellin et al. [31] proposed drilling holes on flank and/or rake faces of turning inserts for delivery of liquid gases in cryogenic turning operations. Wang and Rajurkar [32], Ahmed et al. [33], Venugopal and Chattopadhyay [34], and Wang et al. [35] suggested developing caplets which mount on top of the cutting inserts or modifying cutting tool holder for delivery of liquefied gases in turning operations. Lu et al. [15,36] proposed through the spindle and cutting tool delivery of LN_2_ in milling operations. In their design, LN_2_ is delivered through the spindle into the coolant channels inside a solid-end mill tool. Similarly, Georgiou and Azzopardi [37] patented a through spindle cryogenic cooling tool for boring applications. Various tool designs for delivering cryogens are discussed by Astakhov and Godlevskiy [28].

Reviews of the literature [38,39,40,41] indicate that cryogenic cooling using LN_2_ can improve the machinability of Ti–6Al–4V titanium alloy used in aerospace and medical industries. The improvements are attributed to the increased heat removal during machining and reduced fracture toughness and ductility [42]. Various cutting tool designs were found in the literature for cryogenic machining. These designs concentrated on the delivery of liquid gases such as LN_2_ and CO_2_ into the cutting zone. Currently there is no study on the effect of cutting tool geometries and their relationship specifically for cryogenic machining.

In this paper, the cutting tool geometries of an end mill cutting tool for cryogenic milling of Ti–6Al–4V are investigated. In particular, the rake angle and primary clearance angle are considered for the cutting tool design. The selected cutting tool geometry is further analyzed by changing the cutting speed in an attempt to identify the effect of cutting speed on tool life in cryogenic machining when a specially designed cutting tool is used. A systematic methodology has been developed for machining experiments and the collected data is analyzed and thoroughly discussed.

## 2. Materials and Methods

Titanium alloy, Ti–6Al–4V, is commonly machined using flood cooling. In cryogenic machining, LN_2_ at −197 °C is sprayed into the cutting zone. This would change the temperature of both cemented tungsten carbide cutting tool and the Ti–6Al–4V workpiece material. It is well known that mechanical properties such as material strength, hardness, and toughness are temperature-dependent parameters and they change as the temperature changes [23,43]. Whilst the design of a cutting tool depends on the application, machining operation, and workpiece, the cutting geometries are selected based on material properties of the workpiece and cutting tool. For this research, three rake angles of 10°, 12°, and 14° are selected to be tested using an LN_2_ cryogenic machining environment. The levels were based on preliminary studies and cutting tool manufacturer’s recommendation for machining titanium. The clearance angle affects the friction between the cutting tool and a newly machined surface. Since the cutting edge is the hypothetical point where the rake face and flank face meet, the clearance angle also affects the sharpness and robustness of the cutting edge, and therefore, a correct balance between robustness and sharpness is required when selecting the clearance angle. For this study, the primary clearance angle was tested at two levels of 8° and 10°. The cutting tools were specifically manufactured for this investigation from Extramet EMT 210 solid tungsten carbide with average grain size of 0.8 µm. The tools were coated with average 3 µm TiSiN–TiN Hardcut physical vapor deposition (PVD) coating from IonBond recommended for machining titanium and nickel alloys. Apart from the rake and primary clearance angles, the remaining geometries of the cutting tools were kept constant for this study. The cutting tools had a 12-mm diameter with 3 flutes, 23° second clearance angle, and 250-µm chamfer at 45° for protecting the tool nose. The core diameter of the tools was 7.8 mm with 34°/35° variable helix angle to prevent chatter. In addition, the tools had a 100-µm wiper edge at the minor cutting face to enhance the surface finish of the bottom machined face.

The machining experiments for this investigation was an end milling operation along the length of a Ti–6Al–4V workpiece material with 50 mm x 50 mm x 150 mm dimensions. A new block of workpiece material was used for each machining experiment using a climb milling strategy. The cutting parameters used for experiments are provided in Table 1 based on preliminary studies. The tool overhang was kept constant at 50 mm for all experiments. The tools were balanced at 20,000 rpm. In order to encourage rapid tool wear and limit the experimental time, a high cutting speed of 200 m/min was selected for testing the cutting tool geometries based on previous studies [42,44]. This cutting speed is at the boundary of transition region and high-speed machining for titanium alloys as defined by Schulz and Moriwaki [45].

**Figure 1:**
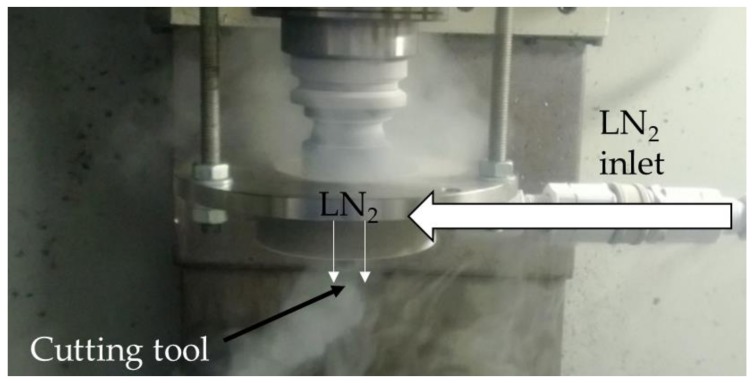
Cryogenic machining setup.

**Figure 2:**
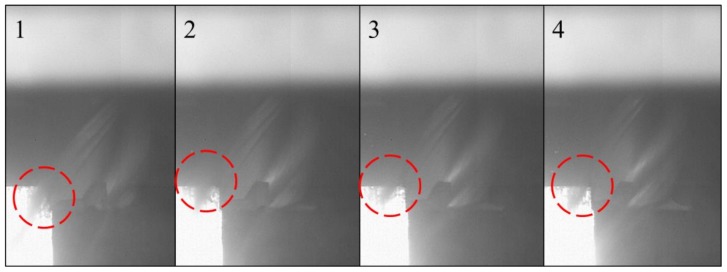
Slow-motion time laps of cryogenic machining showing the delivery of LN_2_ into the cutting zone through the tool’s flute.

All machining experiments were conducted on a VMC XP10 Bridgeport CNC milling center equipped with a retrofittable external cryogenic cooling nozzle. The cryogenic cooling nozzle used for the experiments is shown in Figure 1. The nozzle was placed around the cutting tool covering the periphery of the tool with 1 bar of LN_2_ at 20 kg/h flowrate. There was a 0.5 mm clearance between the nozzle and the cutting tool’s outer diameter. The slow-motion time lapse of the LN_2_ delivered into the cutting zone through the flute of the tool is shown in Figure 2 with the circle highlighting the LN_2_. This figure clearly demonstrates that the LN_2_ was only delivered into the cutting zone without flooding the workpiece. Therefore, the cooling was limited to the cutting zone without significantly affecting the temperature of the workpiece. The tight (0.5 mm) clearance between the nozzle and the cutting tool ensured that LN_2_ can only flow through the cutting tool’s flute into the cutting zone.

In order to measure the tool life for each experiment, the ISO 8688-2 [46] was followed. A maximum flank wear of 300 µm was defined as the end of tool life criterion and other tool wear modes such as notching and chipping were treated as flank wear to identify tool life. The machining experiments were interrupted regularly to measure the tool wear during the experiments. Once the tool life criterion of 300 µm was reached, the tool life was recorded, and the experiment was stopped. The tool wear was measured using a digital optical microscope. In order to ensure the repeatability of the results, the experiments were repeated at least three times. If chipping or notching caused tool failure, the experiment was repeated an extra two times to ensure that the failure was not caused by manufacturing defects.

The surface roughness of the machined samples was measured at the start of each experiment to minimize the effect of tool wear on surface finish. The surface roughness was measured at three points namely, start, middle, and end of the machining path along the length of the workpiece and each measurement was repeated three times.

After investigating the effect of rake and primary clearance angles on tool life and surface roughness, the tool which performed best in terms of tool life and surface finish was selected to analyze the effect of cutting speed on tool life. The cutting speed was varied from 90 m/min to 200 m/min with 10 m/min intervals at 12 levels following a similar procedure as explained above.

## 3. Results

In this section, the results are presented and analyzed. Firstly, the effect of cutting tool geometry on tool life and surface roughness are presented. Based on the results, the optimum cutting geometry is selected for further investigation to study the effect of cutting speed in cryogenic end milling of Ti–6Al–4V titanium alloy.

### 3.1. Cutting Tool Geometry

The effect of rake and primary clearance angle on tool life, tool wear, and surface roughness are shown in this section.

#### 3.1.1. Tool Life

**Figure 3:**
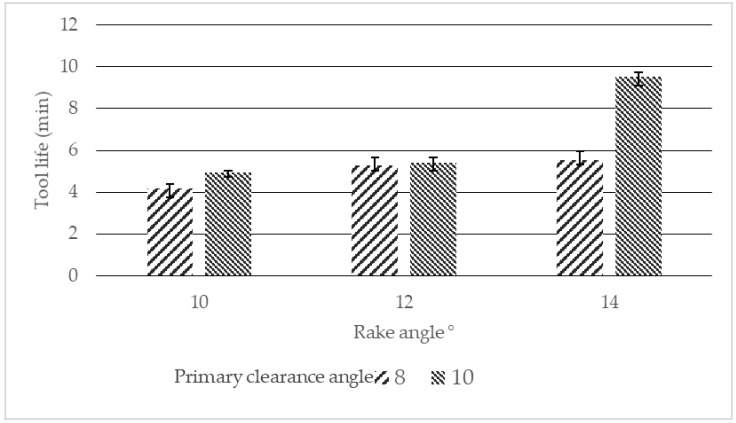
Tool life graph for various rake and primary clearance angles.

A series of machining experiments were conducted by varying rake and primary clearance angles in cryogenic end milling of Ti–6Al–4V as described in Section 2. The tool life for each experiment was recorded and is presented in Figure 3. This shows that increasing the rake angle from 10° to 14° results in increased tool life. The longest tool life of 9.7 min was achieved using the tool with a 14° rake angle and 10° primary clearance angle. In all experiments, the tool with 10° primary clearance angle performed superior to their counterpart with a 8° primary clearance angle. However, the impact of primary clearance angle on tool life was minimal at lower rake angles of 10° and 12°. It is known that increasing positive rake angle can lead to reductions in cutting forces, power consumption, and cutting temperatures [47,48,49,50]. However, after reaching a certain limit, increasing the rake angle will weaken the cutting edge and results in rapid tool wear [48]. In this study, increasing the rake angle from 10° to 14° favorably resulted in increased tool life. Kaymakci et al. [51] reported that increasing clearance angle to an optimum angle results in improved tool life by minimizing flank wear. Further increasing of the clearance angle above the optimum angle, weakens the cutting edge leading to chipping and tool failure.

**Figure 4:**
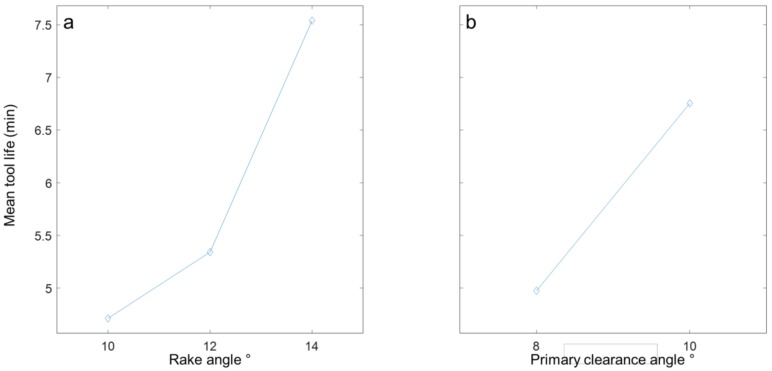
Main effect plots showing the effect of (**a**) rake angle and (**b**) primary clearance angle on tool life.

Analysis of means was also performed on the data for tool life to investigate the effect of rake and primary clearance angle on tool life in cryogenic machining of Ti–6Al–4V as illustrated in Figure 4. The analysis indicated that within the investigation range, increasing the rake angle from 10° to 14° directly results in enhanced tool life irrespective of the primary clearance angle. However, the impact of primary clearance angle is more significant at higher rake angle of 14°. Similarly, as shown in Figure 4, the analysis indicated that 10° primary clearance angle is more suitable when machining Ti–6Al–4V alloy using LN_2_ coolant. Analysis of variance was also performed on the data to identify the significance of rake and primary clearance angles on tool life as provided in Table 2. The analysis indicated that both rake and primary clearance angles have significant effect on tool life with rake angle being the most significant factor.

Since a steady increase in tool life was achieved by increasing the rake angle, a further experiment was conducted using a 16° rake angle. The cutting edge chipped within the first few minutes of the machining experiment and the tool failed catastrophically. As explained above, increasing the rake angle beyond a certain point will result in weakening of the cutting edge and rapid tool wear as reported by Sabberwal and Fleischer [47].

#### 3.1.2. Tool Wear

**Figure 5:**
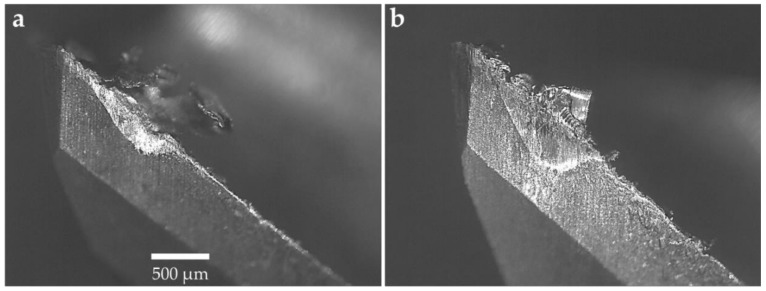
Micrographs of the tool with a 10° rake angle: (**a**) 10° primary clearance angle and (**b**) 8° primary clearance angle.

**Figure 6:**
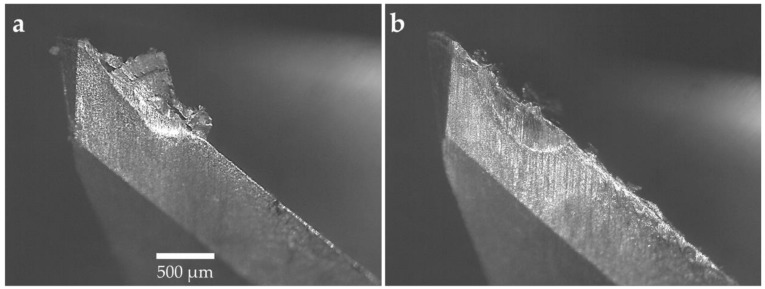
Micrographs of the tool with a 12° rake angle: (**a**) 10° primary clearance angle and (**b**) 8° primary clearance angle.

**Figure 7:**
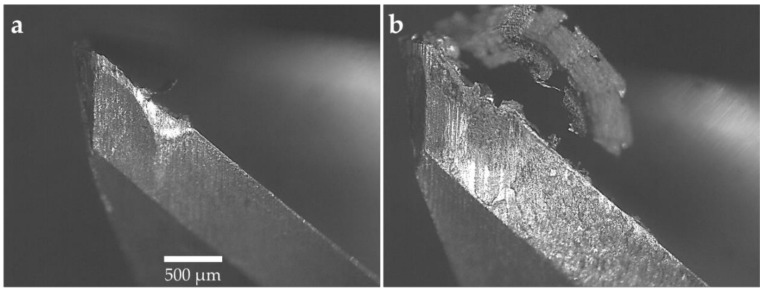
Micrographs of the tool with a 14° rake angle: (**a**) 10° primary clearance angle and (**b**) 8° primary clearance angle.

The machining experiments were interrupted when 300 µm tool wear was reached as per ISO 8688-2 [46]. The cutting tools were analyzed after each machining experiment and the micrographs of the tools were generated using an optical microscope. The micrographs of the tools for 10°, 12°, and 14° rake angles are shown in Figure 5, Figure 6 and Figure 7, respectively. All cutting tools, irrespective of cutting tool geometry, suffered from flank wear and crater wear. Adhesion and abrasion wear were dominant on all cutting tools. Adhesion was in the form of built-up edge and welded chips on the rake face of the tools and smearing on the flank face. The tool wear was initiated by notch wear at the depth of cut followed by adhesion and abrasion on the flank wear leading to tool failure. For all cutting tools, the flank wear was concentrated at the depth of cut as shown in Figure 5, Figure 6 and Figure 7. Figure 7 compares the tool wear of the cutting tools with a 14° rake angle and 8° and 10° primary clearance angles. Whilst there are minimal differences in the abrasive tool wear between the tools with identical rake angles, there is a clear difference in the adhesion wear mechanism particularly on the flank face. The flank face of the tool with a 8° primary clearance angle is fully covered by the Ti–6Al–4V workpiece material beyond the abrasion wear region whilst the adhesion was limited to where the substrate was exposed on the tool with a 10° primary clearance angle. This phenomenon was also observed in tools with 10° and 12° rake angles illustrated in Figure 5 and Figure 6. This is attributed to the fact that at shallow primary clearance angles, the friction between the machined surface and tool flank face is higher. As shown in Figure 6b and Figure 7b, chipping of the cutting edge was identified in machining with a 8° primary clearance angle. In addition, crater wear was noticed on the tools adjacent to the cutting edge on the rake faces of the tool as shown in Figure 6 and Figure 7. The cutting chips adhered onto the rake face of the cutting tool forming a built-up edge (BUE). The BUE was removed by the flow of the chips taking some parts of the coating and cutting tool leaving a crater on the rake face of the tools. The formation of crater weakens the cutting edge leading to chipping and notch wear. This exposed the cutting tool substrate at the cutting edge resulting in accelerated flank wear. Flank wear was the dominant tool wear pattern in all machining experiments.

#### 3.1.3. Surface Roughness

**Figure 8:**
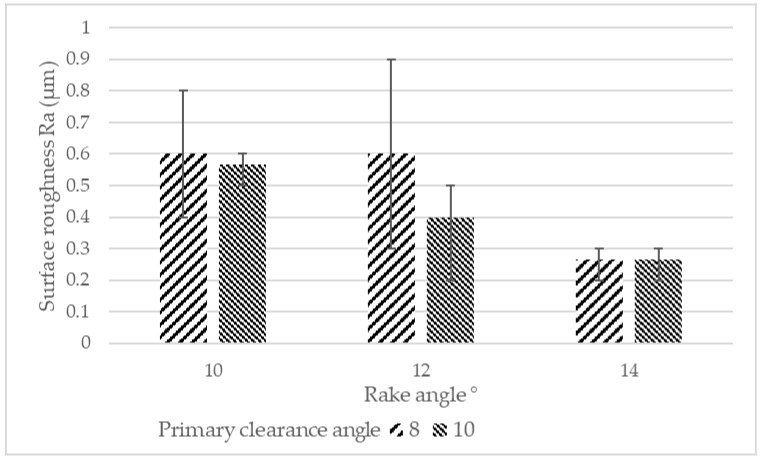
Average surface roughness Ra for tools with different rake and primary clearance angles.

The surface roughness of the workpieces from each machining experiment was measured. The surface roughness measurement was performed at the start of the experiments to minimize the effect of tool wear on surface roughness. The surface roughness was measured at three points namely, start, middle, and end of the machining path and each measurement was repeated three times to ensure repeatability. The average surface roughness Ra from each machining experiment is presented in Figure 8. As shown in the figure, the surface roughness was reduced by increasing both rake angle and primary clearance angle. The lowest surface roughness Ra of 0.27 µm was measured for the tool with a 14° rake angle irrespective of the primary clearance angle. Increasing the primary clearance angle from 8° to 10° in tools with a rake angle of 10° and 12° resulted in improved surface roughness. The highest surface roughness of 0.9 µm was recorded at the end of the machining path for the tool with 12° rake and 8° primary clearance angles. The average surface roughness was 0.6 µm for this experiment. In general, the surface roughness was higher at the exit of the tool for all machining experiments where the tools experiences instability. Analysis of variance indicated that the rake angle was the most significant factor affecting surface roughness Ra with a *p*-value of 0.01.

In machining, the flank face rubs against the newly machined surface when the cutting material causing plastic deformation of the surface. Increasing the primary clearance angle reduces the contact length between the cutting tool and the newly machined surface. Therefore, less plastic deformation takes place on the machined surface leading to improved surface roughness.

### 3.2. Cutting Speed

Based on the analysis in Section 3.1, the tool with a 14° rake angle and a 10° primary clearance angle was selected as the best option within the investigated range for further analysis. One of the major advantages of cryogenic cooling using LN_2_ is that it allows for using higher cutting speeds than flood cooling. Cutting speed is the major contributor to heat generation during machining and is the most dominant parameter affecting the tool life [52]. Effective use of LN_2_ coolant would allow using higher cutting speeds without sacrificing tool life when compared to conventional flood cooling. Therefore, the effect of cutting speed on tool life was investigated as part of this research.

Cutting speeds of 90 m/min to 200 m/min with intervals of 10 m/min (12 levels) were used for machining experiments. The remaining cutting parameters were kept constant at 0.03 mm/tooth feed rate, 1 mm axial depth of cut, and 4 mm radial depth of cut. A series of cutting tools with a 14° rake angle and a 10° primary clearance angle were manufactured from EMT 210 solid tungsten carbide and coated with TiSiN–TiN Hardcut coating. The setup explained in Section 2 was used for the experiments.

**Figure 9:**
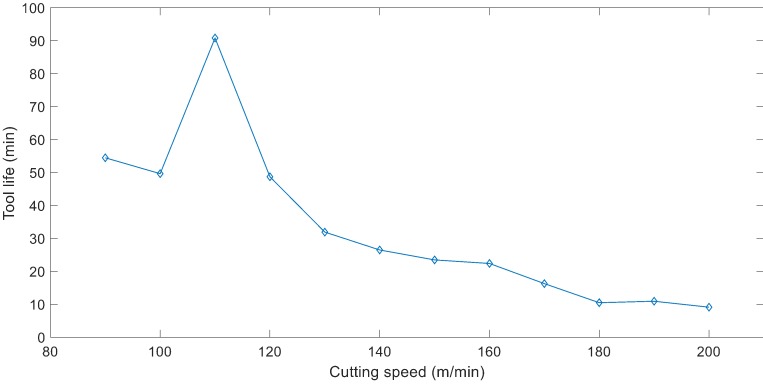
Graph of tool life (min) versus cutting speed.

**Figure 10:**
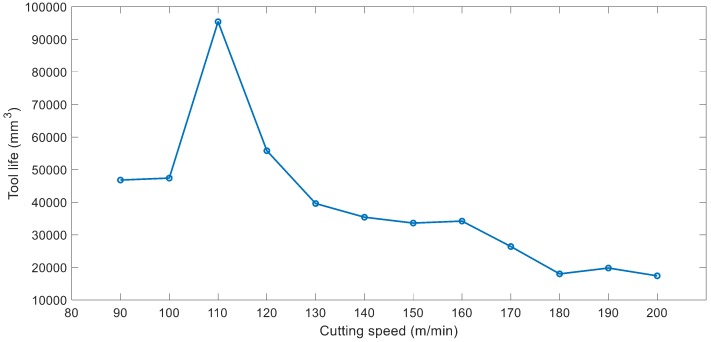
Graph of tool life (mm^3^) versus cutting speed.

The tool life was measured at each cutting speed ranging from 90 m/min to 200 m/min. Following the recommendation from ISO 8688, the results were presented in minutes for tool life as shown in Figure 9. However, as the cutting speed changes, the feed rate also changes. As a result, higher volume of material can be machined at higher speeds within the same time period. Therefore, the second tool life graph based on machined volume was developed as illustrated in Figure 10. Comparing the graphs in Figure 9 and Figure 10, it is clearly shown that whilst tool life in minutes at 90 min/min cutting speed is higher than that of 100 m/min, more material is machined within the tool life of the experiment at 100 m/min cutting speed. Similar comparisons can be made for the tool life results at 100 m/min and 120 m/min cutting speeds.

As shown in Figure 9 and Figure 10, the tool life increases sharply when increasing cutting speed from 100 m/min to 110 m/min. The tool life gradually decreases by increasing cutting speed from 110 m/min to 200 m/min. The longest tool life was recorded to be 91 min equivalent to machining 95400 mm^3^ of Ti–6Al–4V workpiece material achieved at 110 m/min. This constitutes that 10% increase in cutting speed from 100 m/min resulted in 83% increased tool life in minutes and 101% increased material removal.

**Figure 11:**
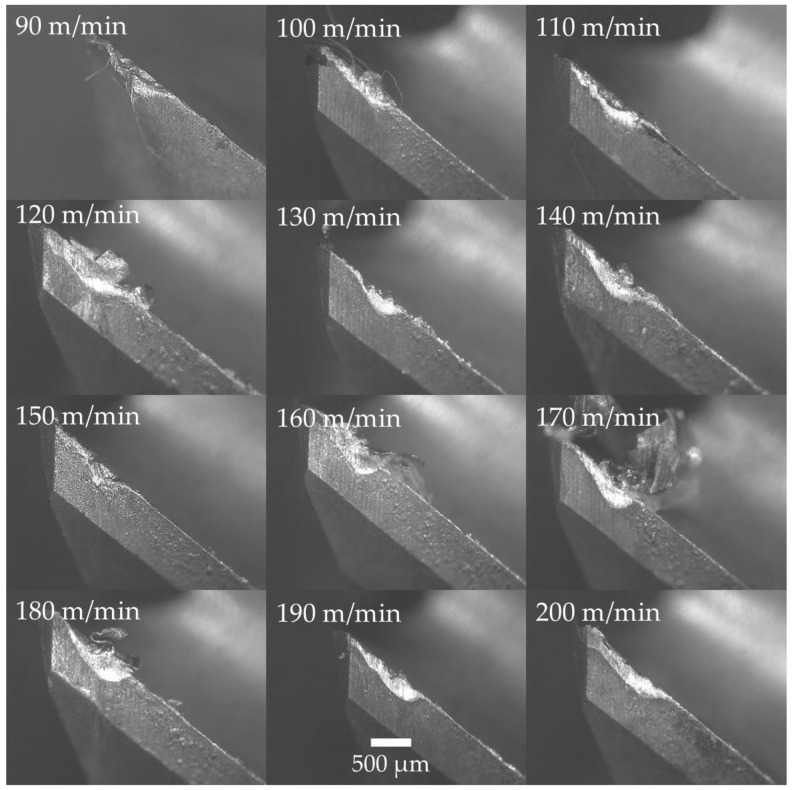
Micrographs of cutting tool wear after machining experiments showing the flank face of the cutting tool.

As shown in Figure 11, the cutting tools suffered mechanical wear such as chipping of the nose and notching at the depth of cut at lower cutting speeds. The tool wear mechanism transformed into thermal wear coupled with adhesion and abrasion at higher cutting speeds. Welding of the chips onto the rake face of the tools was evident in all machining experiments above 100 m/min cutting speed. The tool wear was initiated by removal of the coating and exposing the tungsten carbide substrate. This has resulted in accelerated abrasion and adhesion wear. It is known that titanium is chemically reactive to all known cutting tool materials and the chemical reaction is enhanced at higher temperatures [53]. This is evident by the higher level of adhesion of the workpiece material on both the rake and flank face of the cutting tools from increasing the cutting speed. Shalaby and Veldhuis [54] reported that in machining Ti–6Al–4V with tungsten carbide tools, carbides and oxides of titanium and vanadium workpiece material can form at the cutting zone. These hard materials can act as thermal barriers and solid lubricants at the cutting zone enhancing machining performance at certain cutting speeds. Further investigation is required to confirm this occurrence in cryogenic machining at various cutting speeds.

## 4. Discussion

Exposing the cutting tool and workpiece material to extremely low temperatures (−197 °C) in cryogenic machining impacts on the material properties of the tool and workpiece. The material hardness of both tungsten carbide tool and Ti–6Al–4V increases by reducing the temperature. In addition, the ductility and fracture toughness of Ti–6Al–4V reduces below 100 K [42]. As a result, tungsten carbide cutting tools with geometries optimized for machining Ti–6Al–4V at ambient temperatures does not necessarily perform well in machining at cryogenic conditions.

In this study, three levels of rake angles and two levels of primary clearance angles were investigated. The analysis indicates that increasing the rake angle from 10° to 14° results in a significant improvement in tool life. However, increasing the rake angle from 14° to 16° proved to weaken the cutting edge leading to catastrophic failure of the cutting edge. Nouari and Makich [55] compared the machinability of Ti–6Al–4V with Ti-55531 alloy by varying cutting speed and tool rake angle. In their study, Nouari and Makich [55] used 0° and 20° rake angles tested at 20 m/min, 35 m/min, and 65 m/min. They noted that cutting forces were lower using 20° rake angle whilst the friction coefficient was higher.

All tools suffered notch wear at the depth of cut followed by flank wear concentrated at the depth of cut. A similar observation is reported by Sadik et al. [5] in cryogenic milling where the authors identified notch wear as the dominant tool wear mechanism. Strain hardening of the workpiece material at the depth of cut results in increased workpiece hardness resulting in increased tool wear at the depth of cut. In addition, the cryogenic cooling setup used in this study sprayed LN_2_ along the cutting tool into the cutting zone as well as the top surface of the workpiece. This can result in increased material hardness on the surface of the workpiece leading to increased tool wear at the depth of cut knowing that the hardness of Ti–6Al–4V increases by reducing temperature.

This study showed that using higher rake and primary clearance angles results in improved surface finish from 0.6 µm using the tool with 8° primary clearance and 10° rake angles to 0.26 µm for the tool with 10° primary clearance and 14° rake angles. Higher rake and clearance angles mean sharper cutting edges leading to improved surface finish which is also reported in conventional machining of titanium alloys [55]. In this study, the interactions between the rake and primary clearance angles were not investigated. This will be further investigated in the future.

Cutting speed was identified as the most significant factor affecting the tool life [52,56]. Therefore, cutting speed at 12 levels was investigated using a cutting tool with 14° rake and 10° primary clearance angle. The analysis indicated that the tool life increases by increasing cutting speed from 100 m/min to 110 m/min. The tool life takes a downward slope when cutting speed is further increased as demonstrated in Figure 10. According to Astakhov [57], in machining operations, there is an optimum cutting temperature which yields to minimum tool wear and cutting forces and high-quality surface finish. “This temperature is invariant to the way it has been achieved” (p. 641, [57]). In this research, the combination of heat generation through increased cutting speed and heat removal through cryogenic cooling has resulted in the longest tool life of 91 min at 110 m/min cutting speed. The measurement of cutting temperature was beyond this investigation which will be further studied. Ti–6Al–4V alloy is commonly machined at cutting speeds ranging from 60 m/min to 80 m/min with solid carbide tools using conventional flood cooling [55,58,59]. Based on this, up to 83% increased cutting speed and therefore material removal rate is achieved by using cryogenic cooling and the new proposed cutting tool geometry.

## 5. Conclusions

A comprehensive review of literature in cryogenic machining revealed that there is a significant need for investigating the effect of various cutting tool geometries in cryogenic end milling of Ti–6Al–4V titanium alloy. This study identified that:

The material properties of workpiece and cutting tool are affected by cryogenic cooling. A new cutting tool with improved geometries was generated to improve tool life and surface roughness in cryogenic machining of Ti–6Al–4V. The new cutting tool can withstand machining at 200 m/min cutting speed for over 9 min.

This new cutting tool was used for machining at various cutting speeds. The investigations indicated that the balance between heat generation and heat dissipation at the cutting zone are most favorable at 110 m/min cutting speed yielding to 91 min tool life equivalent to 95,400 mm^3^ of machined material volume.

This research has shown that the application of cryogenic cooling using LN_2_ together with the proposed cutting tool has a significant effect in the finish machining of Ti–6Al–4V titanium alloy used in aerospace and medical industries, resulting in up to 83% increased material removal rate and improved productivity.

## Figures and Tables

**Table 1:** Cutting parameters used for testing cutting tool rake angle and primary clearance angle.

Cutting speed	Chip load	Axial depth of cut	Radial depth of cut
200 m/min	0.03 mm/tooth	1 mm	4 mm

**Table 2:** ANOVA of the results for tool life based on rake and primary clearance angles.

Source	Sum of squares	Degree of freedom	Mean square	P
Rake angle	28.72	2	14.36	0.0004
Primary clearance angle	11.60	1	11.60	0.0045
Error	14.26	14	1.02	
Total	54.58	17		
